# Clinical characteristics of Egyptian male patients with COVID‐19 acute respiratory distress syndrome

**DOI:** 10.1371/journal.pone.0249346

**Published:** 2021-04-16

**Authors:** Ahmed S. Doghish, Walid F. Elkhatib, Essam A. Hassan, Ahmed F. Elkhateeb, Eman E. Mahmoud, Mona I. Ahmed, Mahmoud A. F. Khalil

**Affiliations:** 1 Department of Biochemistry, Faculty of Pharmacy (Boys), Al-Azhar University, Nasr City, Cairo, Egypt; 2 Department of Biochemistry, Faculty of Pharmacy, Badr University in Cairo (BUC), Badr City, Cairo, Egypt; 3 Microbiology and Immunology Department, Faculty of Pharmacy, Ain Shams University, African Union Organization St., Abbassia, Cairo, Egypt; 4 Department of Microbiology and Immunology, Faculty of Pharmacy, Galala University, New Galala City, Suez, Egypt; 5 Department of Tropical Medicine, Faculty of Medicine, Fayoum University, Fayoum, Egypt; 6 Department of Critical Care Medicine, Faculty of Medicine, Fayoum University, Fayoum, Egypt; 7 Department of Clinical and Chemical Pathology, Faculty of Medicine, Fayoum University, Fayoum, Egypt; 8 Department of Chest Diseases, Faculty of Medicine, Fayoum University, Fayoum, Egypt; 9 Department of Microbiology and Immunology, Faculty of Pharmacy, Fayoum University, Fayoum, Egypt; University Magna Graecia of Catanzaro, ITALY

## Abstract

**Background:**

Coronavirus disease 2019 (COVID-19) is a serious illness caused by severe acute respiratory syndrome coronavirus 2 (SARS-CoV-2) and in severe cases associated with acute respiratory distress syndrome (ARDS).

**Objective:**

To describe the clinical characteristics of patients with ARDS-COVID-19.

**Materials and methods:**

This study involved 197 male Egyptian participants, among them111 COVID-19 patients presented with ARDS, 60 COVID-19 patients presented with non-ARDS, and 26 Non-COVID-19 patients. We reported the analysis results of clinical and laboratory information, including blood routine tests, blood biochemistry parameters [aspartate aminotransferase (AST), alanine aminotransferase (ALT), creatinine and C‐reactive protein (CRP)], thrombotic activity (D‐dimer) and serum ferritin and lactate dehydrogenase (LDH).

**Results:**

The levels of hemoglobin, AST, creatinine, monocyte count, monocyte %, RBC count, TLC, and platelet count were not significantly different among the groups. The lymphopenia and increased CRP, ALT, D-dimer, ferritin, and LDH were observed in patients with ARDS-COVID-19.

**Conclusion:**

COVID-19 patients with ARDS presented with lymphopenia, increased thrombotic activity, increased CRP, LDH, and ferritin levels. The results revealed that CRP, D-dimer, LDH levels, and lymphopenia have a significant association with the COVID-19 severity and can be used as biomarkers to predict the disease severity.

## Introduction

A novel coronavirus, coronavirus disease 2019 (COVID-19), was first recognized in Wuhan (the capital of Hubei Province), China, in December 2019 [1–3]_._ The main routes of virus transmission between people are contact routes and respiratory droplets [4]. In this disease, most patients develop mild respiratory tract infection symptoms, with some people rapidly developed acute respiratory distress syndrome (ARDS) that requires hospitalization, respiratory support, and oxygen therapy [5]. Criteria for ARDS diagnosis include a progression of pulmonary findings, and bilateral pulmonary infiltrates on chest imaging, absence of cardiogenic respiratory failure, and profound hypoxemia [6]. Patients with COVID‐19 ARDS have inflammatory lung, mucus production in the airways, higher proinflammatory cytokines levels, and pulmonary microthrombosis [7]. The diagnosis, control, prevention, and treatment are very critical subjects in COVID-19. So, early diagnosis and effective and timely treatment are very crucial. Delayed diagnosis of COVID-19 leads to a rapid spread of infection with increased risk for ARDS [8]. In the current study, we investigated hematological and coagulation characteristics in Egyptian patients. Moreover, the predictive values and diagnostic efficacy of clinical laboratory data were analyzed for ARDS-COVID-19 patients.

## Methods

### Patients and study design

This retrospective study was conducted on male Egyptian participants with or without COVID-19 at Fayoum University Hospital (Fayoum, Egypt), from May 11, 2020, to June 18, 2020. One hundred and ninety-seven male participants aged 22 to 75 years, with a median age of 40 years (interquartile range [IQR], 30–50), were participated in this study. We considered male subjects only to neglect the effect of fluctuation of female’s menstrual cycle that may affect some of the clinical laboratory tests as creatine kinase, c-reactive protein, white blood cell, and others [9]. Of 197 suspected male cases, 171 (86.8%) patients were positive for COVID-19, and 26 (13.2%) were negative. Nasopharyngeal swabs were collected by a health care professional and tested for COVID-19 by real-time reverse transcription-polymerase chain reaction (rRT-PCR). The COVID-19 patients were divided into two groups, comprising 111 (64.9%) patients diagnosed with ARDS and 60 (35.1%) with non-ARDS. Blood samples from each adult male patient were collected and used for hematological investigations.

This study was performed in accordance with the ethical principles of the 1975 Declaration of Helsinki and approved by the Ethics Committee of Fayoum University Hospital, and written informed consent was obtained from each patient before clinical and laboratory investigations for the involvement of anonymous patients’ data in scientific research for the current retrospective study.

### Data collection

Clinical and laboratory information, including blood routine tests [hemoglobin, red blood cell (RBC) count, total leukocyte count (TLC), lymphocyte count, lymphocyte %, monocyte count, monocyte%, neutrophil %, and platelet count], blood biochemistry parameters [aspartate aminotransferase (AST), alanine aminotransferase (ALT), creatinine, and C‐reactive protein (CRP)], thrombotic activity (D‐dimer), serum ferritin, and lactate dehydrogenase (LDH) from 197 male Egyptian patients with or without COVID-19 were collected from electronic patients’ medical records using standardized forms.

### Statistical analysis

All statistical analyses were performed using GraphPad Prism (GraphPad Prism 8.0 Software, San Diego, CA, USA). All data were presented as median and IQR. For comparing more than two groups, both one-way ANOVA and Tukey’s multiple comparisons post-hoc test were used while Student’s t-test was used, if two different groups were compared. Differences were considered significant at *P*-value < 0.05. The diagnostic potential was compared among tested parameters using receiver operating characteristic (ROC) curve analysis.

## Results

### Baseline characteristics

The study involved 197 male Egyptian participants with or without COVID-19. Among them 111 COVID-19 patients who presented with ARDS; their median age was 39.50 years (IQR, 29.5–55), 60 COVID-19 patients who presented with non-ARDS; their median age was 41 years (IQR, 30–50) and 26 Non-COVID-19 participants; their median age was 42 years (IQR, 31.5–55). There was no significant difference between the three groups on age (*p* > 0.05).

The common symptoms of the COVID-19 patients at the onset of sickness were fever (147 [85.96%]), dry cough (112 [65.49%]), fatigue (70 [40.39%]), dyspnea (125 [73.09%]), and myalgia (71 [41.52%]); less common symptoms were diarrhea, nausea, headache, vomiting, loss of smell, loss of taste and sore throat (Table 1).

**Table 1:** Baseline characteristics of patients with or without COVID-19.

Item	Suspected Non-COVID-19	Total COVID-19	Non-ARDS-COVID-19	ARDS-COVID-19
	N (26)	N (171)	N (60)	N (111)
Age, median (IQR), years	42 (31.5–55)	40 (29.75–51.25)	41 (30–50)	39.5 (29.5–55)
Signs and symptoms n (%)				
Fever	2 (7.69)	147 (85.96)	46 (76.66)	101 (90.99)
Dry cough	2 (7.69)	112 (65.49)	14 (23.33)	98 (88.28)
Fatigue	1 (3.85)	70 (40.93)	23 (38.33)	47 (42.34)
Myalgia	0	71 (41.52)	23 (38.33)	48 (43.24)
Dyspnea	2 (7.69)	125 (73.09)	22 (36.66)	103 (92.79)
Diarrhea	2 (7.69)	33 (19.29)	12 (20)	21 (18.91)
Nausea	0	24 (14.04)	8 (13.33)	16 (14.41)
Headache	0	38 (22.22)	11 (18.33)	27 (24.32)
Vomiting	0	22 (12.86)	8 (13.33)	14 (12.61)
Loss of Smell	1 (3.85)	44 (25.73)	10 (16.66)	34 (30.63)
Loss of taste	1 (3.85)	44 (25.73)	10 (16.66)	34 (30.63)
Sore throat	1 (3.85)	50 (29.23)	11 (18.33)	39 (35.13)

ARDS: acute respiratory distress syndrome, IQR: interquartile range. Data are represented as median (IQR) or n (%)

### Clinical laboratory data

The hematological features of the patients are shown in Table 2. The levels of hemoglobin, AST, creatinine, monocyte count, monocyte %, RBC count, TLC, and platelet count were not significantly different (*p* > 0.05) among the groups. The level of CRP (mg/L) was significantly higher in the ARDS-COVID-19 group [40.3 (6.05–72.60)] when compared to the non-ARDS-COVID-19 [12.30 (5.00–48.00)] and suspected non-COVID-19 [4.330 (1.67–10.90)] groups. Overall, there is a significant (*p* < 0.05) increase in CRP level [25.35 (5.22–66)] in total COVID-19 positive patients as compared to the non-COVID-19 group.

**Table 2:** Clinical laboratory data of patients with or without COVID-19.

item	Suspected Non-COVID-19	Total COVID-19	Non-ARDS-COVID-19	ARDS-COVID-19
	N (26)	N (171)	N (60)	N (111)
Hemoglobin (g/dL)	13.50 (12.10–15.05)	13.2 (12.1–14.6)	12.60 (11.43–14.30)	13.40 (12.30–14.70)
CRP (mg/L)	4.330 (1.67–10.90)	25.35 (5.22–66) *	12.30 (5.00–48.00)	40.30 (6.05–72.60) *^,^ **
ALT (U/L)	20 (12–31.75)	34.6 (21.68–65) *	29.45 (19–40.2)	38.45 (22.93–73.38) *^,^ **
AST (U/L)	18.50 (15.5–28)	35 (24–49) *	32.5 (20.25–41)	36 (25–49)
Creatinine (mg/dL)	0.9 (0.87–0.98)	1 (0.7–1.25)	0.9 (0.79–1.3)	1 (0.7–1.27)
D-dimer (mg/L)	0.4 (0.27–0.5)	2.5 (1–7.25) *	0.7 (0.50–1.7)	3.66 (0.55–8) *^,^ **
Ferritin (ng/mL)	134.1 (43.15–160)	257.5 (118–657.9) *	244 (72.18–382)	301.4 (134.3–978) *^,^ **
LDH (U/L)	171 (161–224)	313 (233–452) *	236 (209–366)	331.5 (241.5–521.5) *^,^ **
Lymphocyte %	39.90 (23.45–48.9)	31.8 (14–41.48) *	34.50 (22.58–43.35)	25.00 (12–40) *^,^ **
Lymphocyte count (per μL)	2005 (1540–2825)	1501 (928.3–2060) *	1850 (1315–2323)	1342 (760–1906) *^,^ **
Monocyte count (per μL)	440 (297.5–702.5)	470 (313.5–627)	415 (297.5–511)	486 (350–671)
Monocyte%	9.05 (4.07–10.63)	8.7 (6–11.1)	7 (5–10.13)	9 (6.4–12)
RBC Count (Millions/cmm)	4.95 (4.32–5.42)	4.79 (4.43–5.23)	4.79 (4.39–5.18)	4.8 (4.51–5.26)
TLC (thousands/cmm)	5.8 (4.67–7.53)	5.53 (4–7.8)	5.55 (3.5–8.1)	5.56 (4.3–7.58)
Neutrophil %	47.5 (37.35–62.1)	57.45 (43.9–73.05)	51.9 (40.95–67.23)	61.5 (47–78) *^,^ **
Platelet count (thousands/cmm)	248 (208–289)	236 (180.8–294.5)	225 (183.3–290.8)	238 (177–295.8)

ARDS: acute respiratory distress syndrome, CRP: C-reactive protein, ALT: alanine aminotransferase, AST: aspartate aminotransferase, RBC: red blood cell, TLC: leukocyte count, IQR: interquartile range. Data are represented as median (IQR) or n (%),

*Significantly different from suspected Non-COVID-19 subjects at *p* < 0.05,

**Significantly different from non-ARDS-COVID-19 subjects at *p* < 0.05.

The patients infected with COVID-19 showed significant (*p* < 0.05) increased ALT (U/L) [34.6 (21.68–65)] and lymphopenia [1501 (928.3–2060)] when compared to non-COVID-19 group. The level of ALT (U/L) was significantly higher in the ARDS-COVID-19 group [38.45 (22.93–73.38)] than in the non-ARDS-COVID-19 [29.45 (19–40.2)] and suspected non-COVID-19 [20 (12–31.75)] groups. The lymphocyte count (per μL) was significantly lower in the ARDS-COVID-19 group [1342 (760–1906))] when compared to non-ARDS-COVID-19 [1850 (1315–2323))] and suspected non-COVID-19 [2005 (1540–2825)] groups.

COVID-19 positive patients showed significant increased D-dimer (mg/L) [2.5 (1–7.25)] and ferritin (ng/mL) [257.5 (118–657.9)] when compared to non-COVID-19 group. The D-dimer (mg/L) and ferritin (ng/mL) levels were significantly (*p* < 0.05) higher in the ARDS-COVID-19 group [3.66 (0.55–8) and 301.4 (134.3–978), respectively] than in the non-ARDS-COVID-19 [0.7 (0.50–1.7)] and 244 (72.18–382), respectively] and suspected non-COVID-19 [0.4 (0.27–0.5) and 134.1 (43.15–160), respectively] groups.

The level of LDH (U/L) was significantly (*p* < 0.05) higher in the ARDS-COVID-19 [331.5 (241.5–521.5)] when compared to non-ARDS-COVID-19 groups [236 (209–366)] and suspected non-COVID-19 [171 (161–224)] groups.

### ROC curve analysis

ROC curves were used to evaluate the diagnostic efficiency of CRP, D-dimer, ferritin, and LDH levels. The total accuracy and sensitivity of CRP, D-dimer, ferritin, and LDH at the selected cutoff values are in Table 3. The sensitivity of CRP, D-dimer, ferritin, and LDH were 74.38%, 80.0%, 62.62% and 76.42%, respectively.

**Table 3:** Diagnostic efficacy for CRP, D.dimer, ferritin, and LDH levels among studied groups.

	CRP	D.dimer	Ferritin	LDH
AUC	0.80	0.79	0.72	0.89
95% CI (SE)	0.718–0.891 (0.0458)	0.689–0.897 (0.0531)	0.623–0.816 (0.0492)	0.807–0.980 (0.044)
Sensitivity	74.38%	80%	62.62%	76.42%
Specificity	65.38%	68.75%	86.36%	90.91%
Cutoff	5.8	0.49	167.9	230.5
P-value	<0.0001	0.0003	0.0013	<0.0001
PPV	92.97	91.23	95.38	98.95
NPV	29.31	45.83	33.93	25.64
Total accuracy	73.19	77.78	66.94	77.61

AUC: area under the receiver operating characteristic curve, CI: confidence interval, SE: Standard error, PPV: positive predictive value, NPV: negative predictive value, CRP: C‐reactive protein, LDH: lactate dehydrogenase.

**Fig 1:**
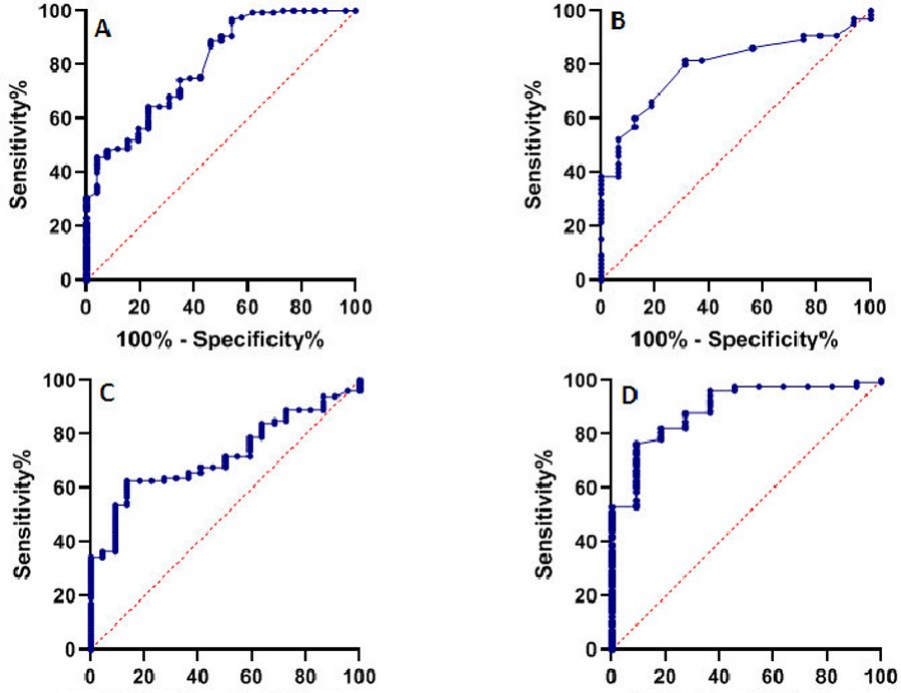
ROC curve for CRP (A), D.dimer (B), ferritin (C), and LDH (D) levels among investigated groups.

As shown in Table 3 and Fig 1, CRP area under the ROC curve (AUC) = 0.80, 95% CI: 0.718–0.891, P <0.0001; D-dimer AUC = 0.79, 95% CI: 0.689–0.897, P = 0.0003; ferritin AUC = 0.72, 95% CI: 0.623–0.816, P = 0.0013; LDH AUC = 0.89, 95% CI: 0.807–0.980, P <0.0001. AUC more than 0.75 represents a good diagnostic value.

## Discussion

Coronavirus disease 2019 is a serious illness caused by severe acute respiratory syndrome coronavirus 2 (SARS-CoV-2) [10]. In severe cases of COVID-19, ARDS is the most serious complication [11]. This may be due to a diversity of mechanisms, including neutrophil activation [12], hypercytokinemia [13], and the renin-angiotensin system dysregulation [14].

In the current study, we reported the analysis results of blood routine, blood biochemistry parameters, thrombotic activity, serum ferritin, and lactate dehydrogenase of 197 Egyptian male patients tested for coronavirus by rRT-PCR after being admitted to the Fayoum University Hospital emergency room as suspected COVID-19 cases. Of them, 171 tested positive, whereas 26 tested negatives.

The results revealed that TLC, monocyte count, monocyte%, hemoglobin, and RBC count were not significantly different between the ARDS-COVID-19 group and the Non-ARDS-COVID-19 group. The lymphocyte count was significantly lower in the ARDS-COVID-19 group when compared to non-ARDS-COVID-19 and suspected non-COVID-19 groups. In accordance with Huang et al. [7] who described low lymphocyte counts in most patients. Lymphopenia is a prominent characteristic of critically ill patients with COVID-19, resulting from the direct destruction of lymphocytes, especially CD4^+^ T and CD8^+^ T cells [15], or cytokine-mediated lymphocyte destruction [16].

Conversely, Gao et al. [1] found that lymphocyte count was not significantly different between patients with severe and mild COVID-19, and this may be attributed to variability in the population, gender difference as the current study selected male subjects while the Chinese one uses data of both male and female patients, and sample size (Gao et al. [1] study involved smaller sample size).

This study revealed that thrombotic activity (D‐dimer) was significantly elevated in ARDS-COVID-19 group [3.66 (0.55–8) mg/L] when compared to non-ARDS-COVID-19 group [0.7 (0.50–1.7) mg/L] and suspected non-COVID-19 group [0.4 (0.27–0.5) mg/L]. Higher D-dimer levels in COVID-19 patients with severe disease have been reported in China [17]. Gao et al. [1] found a significant difference in D‐dimer level between patients with severe and mild COVID-19, which suggests that elevated D-dimer levels have a significant association with the COVID-19 severity. Our results revealed that COVID-19 patients with ARDS would have abnormal coagulation, and the acute lung inflammatory response may have been associated with increased thrombotic activity [18].

This study demonstrated that the median platelet counts in the ARDS-COVID-19 group were within the normal range. This result is in agreement with a previous study in China [19]. On the other hand, Yin et al. [20] reported a significant elevation in platelet count in patients with severe pneumonia induced by COVID-19 than in those induced by non-COVID-19. There was no significant difference (p > 0.05) regarding kidney functions among the groups. On the other hand, ALT showed a significant difference (p < 0.05) between the ARDS-COVID-19 group and the non-ARDS-COVID-19 group.

In this study, COVID-19 patients showed elevated LDH, ferritin, and CRP levels compared to the suspected non-COVID-19 group. Huang et al. and Wang et al. [7, 21] found LDH level was significantly elevated in COVID-19 cases, which required intensive care unit (ICU) care compared to cases that did not require ICU care in China. Severe COVID-19 infections may cause cytokine-mediated lung tissue damage and LDH enzyme release [22]. Since LDH isoenzyme-3 (LDH-3) is present in lung tissue, patients with severe ARDS can be expected to release LDH in greater amounts in the circulation.

Statistical analysis in this study showed that the ferritin level was significantly (p < 0.05) elevated in ARDS-COVID-19 group [301.4 (134.3–978) ng/mL] when compared to non-ARDS-COVID-19 group [244 (72.18–382) ng/mL] and suspected non-COVID-19 group [134.1 (43.15–160) ng/mL]. Our data confirm that increased ferritin level was directly associated with the disease severity (Table 2). This result was in agreement with Lin et al. [23], who reported an increase of ferritin level in Chinese patients with severe COVID-19 than patients with non-severe COVID-19 disease. Raised ferritin levels in circulation could indicate severe inflammatory reaction in ARDS-COVID-19, and elevated ferritin levels in circulation may play an important role by contributing to cytokine storm development resulting in pulmonary edema and ARDS [24].

Results of the current study revealed a positive association between CRP level and lung disorders in COVID-19 patients. This result was in agreement with Wang [25], who reported an increase in CRP level in Chinese patients with critical and severe COVID-19 than patients with moderate and mild COVID-19.

ROC curves were used to evaluate the sensitivity and specificity of different biomarkers in ARDS-COVID-19 patients. The AUCs of CRP, D-dimer, and LDH were 0.80, 0.79, and 0.89, respectively, while the AUC of ferritin was 0.72, below 0.750, unlikely to be an acceptable predictive value. The results revealed that the levels of CRP, D-dimer, and LDH, AUC more than 0.75, were valuable diagnostic biomarkers for distinguishing ARDS-COVID-19 from non-ARDS-COVID-19.

## Conclusion

COVID-19 Egyptian patients with ARDS showed lymphopenia, increased thrombotic activity, elevated CRP, LDH, and ferritin levels. Furthermore, elevated CRP, D-dimer, and LDH levels as well as lymphopenia have a significant association with the COVID-19 severity and can be used as valuable biomarkers to predict the disease severity.
