# Correction: Clinical characteristics of Egyptian male patients with COVID‐19 acute respiratory distress syndrome

**DOI:** 10.1371/journal.pone.0259432

**Published:** 2021-10-27

**Authors:** Ahmed S. Doghish, Walid F. Elkhatib, Essam A. Hassan, Ahmed F. Elkhateeb, Eman E. Mahmoud, Mona I. Ahmed, Mahmoud A. F. Khalil

The ORCID iD is missing for the second corresponding author. Please see the author’s ORCID iD here:

- Author Walid F. Elkhatib’s ORCID iD is: 0000-0001-5815-3200 (https://orcid.org/0000-0001-5815-3200).
